# Supplementary material for: Knockdown of DJ-1 Resulted in a Coordinated Activation of the Innate Immune Antiviral Response in HEK293 Cell Line
Source: Int J Mol Sci. 2024 Jul 10;25(14):7550. doi: 10.3390/ijms25147550 (PMC11277157; doi:10.3390/ijms25147550)
Supplement: Supplementary file 1 [file ijms-25-07550-s001.zip › Supplementary Figures S1-S3.pdf]

Supplementary Figures

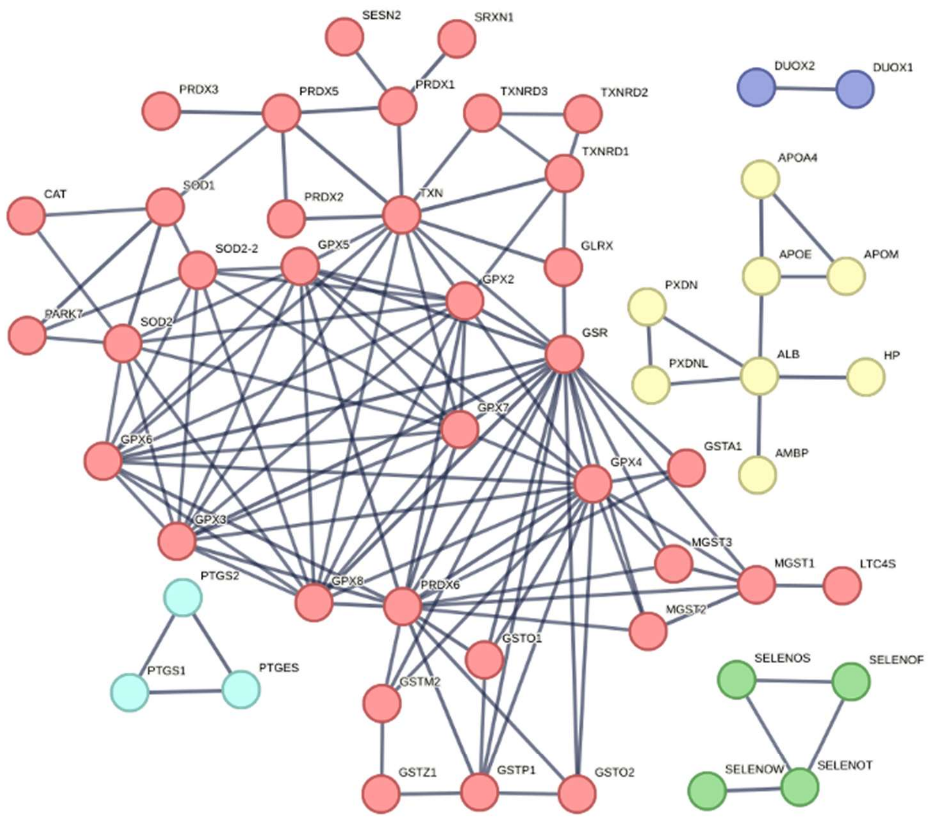

bubble	cluster Id	gene count	protein names
	Cluster 1	35	CAT, GLRX, GPX2, GPX3, GPX4, GPX5, GPX6, GPX7, GPX8, GSR, GSTA1, GSTM2, GSTO1, GSTO2, GSTP1, GSTZ1, LTC4S, MGST1, MGST2, MGST3, PARK7, PRDX1, PRDX2, PRDX3, PRDX5, PRDX6, SESN2, SOD1, SOD2, SOD2-2, SRXN1, TXN, TXNRD1, TXNRD2, TXNRD3
	Cluster 2	8	ALB, AMBP, APOA4, APOE, APOM, HP, PXDN, PXDNL
	Cluster 3	4	SELENOF, SELENOS, SELENOT, SELENOW
	Cluster 4	3	PTGES, PTGS1, PTGS2
	Cluster 5	2	DUOX1, DUOX2

Figure S1. Gene set of antioxidant activity (76 genes, GO: 0016209). A STRING-based network of the connected set of "antioxidant activity" (STRING PPI score >0.9). The genes are partitioned into 5 colored groups (Top), with a dominant cluster (red; 35 genes). A full list is all listed genes is in Supplementary Table S1.

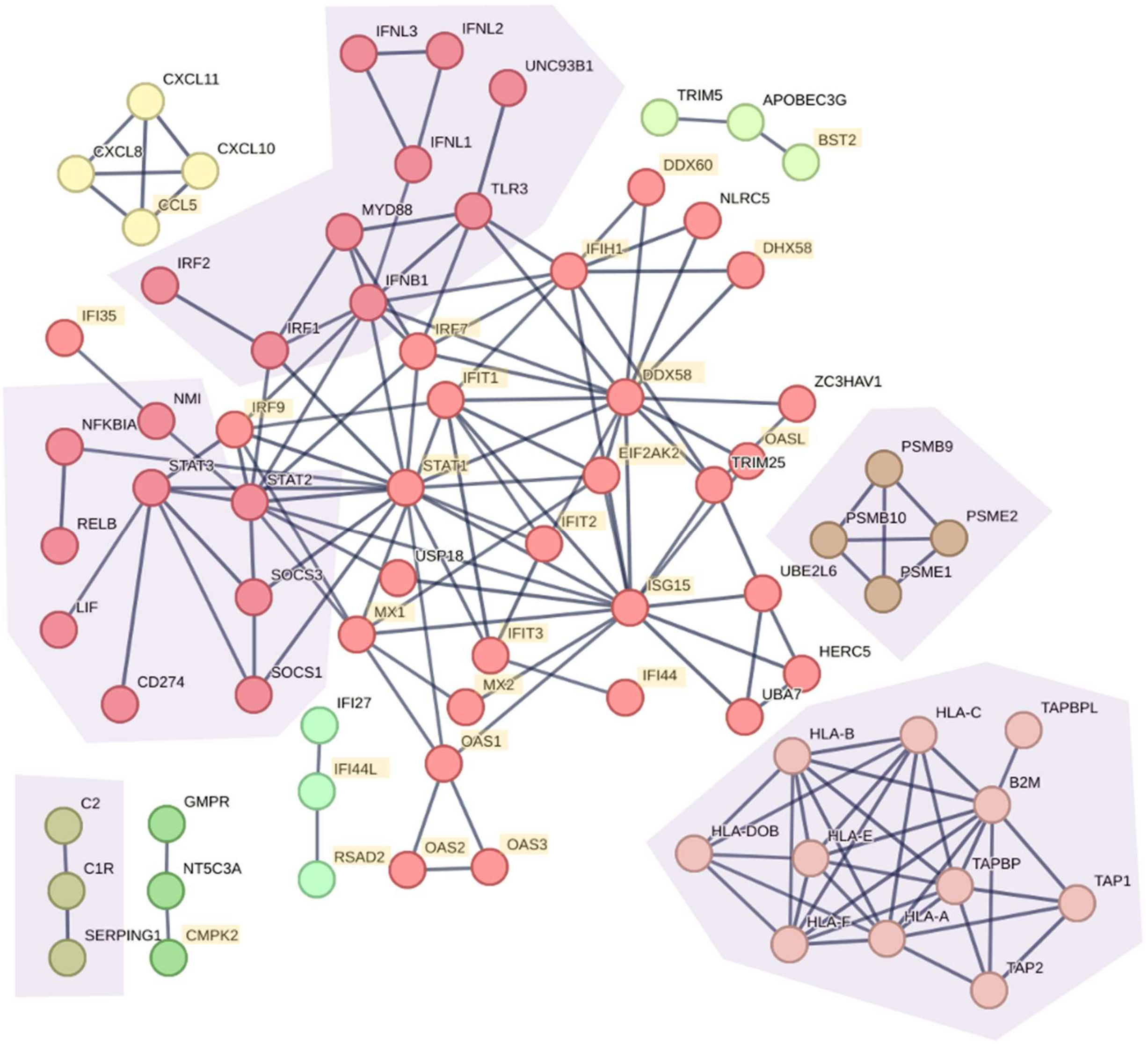

Figure S2. Gene set of upregulated DEG of siRNA for DJ-1 versus the siRNA used for the negative control (esiRNA RULC, see Methods). A STRING-based network of the connected set is of high confidence (STRING PPI score >0.9) and using only three sources of evidence: text mining, databases and experiments (excluded co-expression, co-occurrences, neighborhood, gene fusion). There are 8 subnetworks with at least 3 nodes each. The gene symbols annotated as upregulated by siRNA RULC are indicated by yellow frames. The rest are genes that were only induced at the KD of DJ-1 cells. The subnetworks that enriched with DJ-1 induced gene set are highlighted with a purple background.

A full list is all listed genes is in Supplementary Table S2. The DEG analysis for each cellular setting, is s available in Table S3.

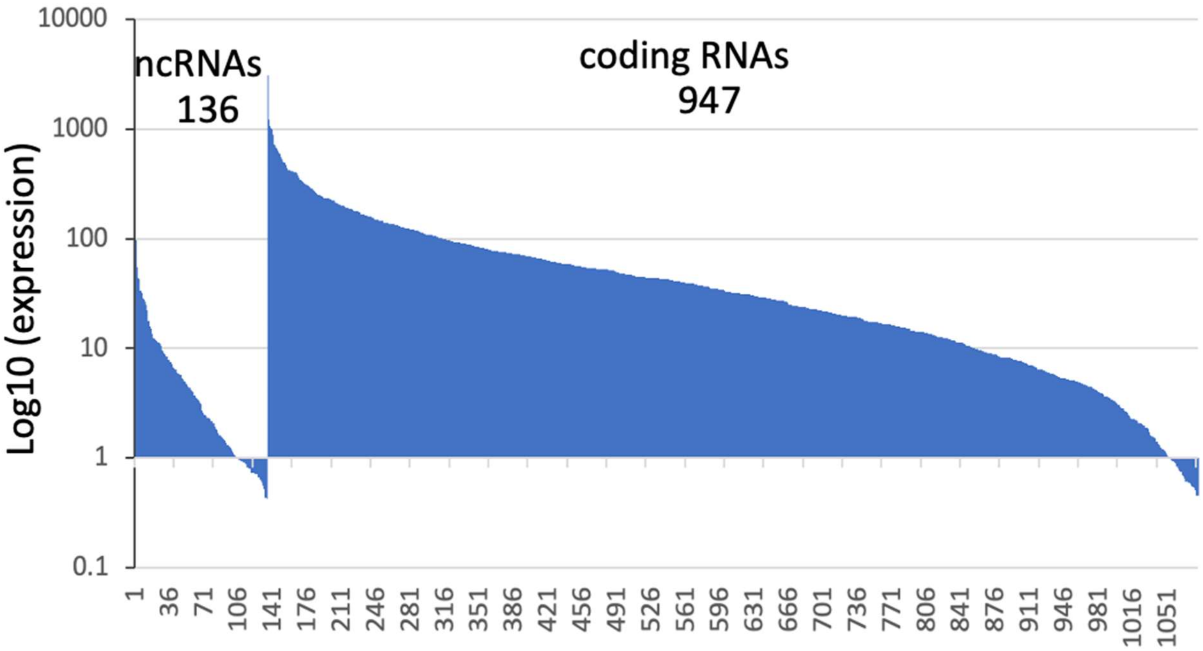

Figure S3. Ranking of the expression levels of 1083 DEG that are statistically significant (FDR<0.05) between KD DJ-1 and non-treated (NT) cells. Genes were partitioned to 136 ncRNAs (1-136), and additional 947 coding RNAs. The expression levels were normalized by TMM where all identified 18,158 transcripts were normalized to 1 million (for a complete gene list see Supplementary Table S2).
